# Associations between psychological distress in adolescence and menstrual symptoms across life: Longitudinal evidence from the 1970 British Cohort Study

**DOI:** 10.1016/j.jad.2024.03.069

**Published:** 2024-06-01

**Authors:** C. Martins, J.J. Mitchell, M. Hamer, J.M. Blodgett

**Affiliations:** a Institute of Sport, Exercise & Health, Division of Surgery and Interventional Science, UCL, London, UK; b Faculty of Mathematical and Physical Sciences, UCL, London, UK; c University College London Hospitals NIHR Biomedical Research Centre, London, UK

**Keywords:** Psychological distress, Menstrual symptoms, Reproductive health, Life course, Risk prediction, Adolescent health, Genecology

## Abstract

**Purpose:**

This study aimed to investigate the association between psychological distress (PD) at age 16 and menstrual symptoms experienced across women's life.

**Methods:**

Up to 2584 females from the 1970 British Cohort Study, a study of individuals born within one week in 1970, were included. PD at age 16 was measured with the 12-item General Health Questionnaire. Three categories were derived: low PD (<11), moderate PD (11–15), and severe PD (>15). Five menstrual health symptoms were self-reported at each age (16, 30 and 42 years). Binomial logistic regressions examined associations between PD at age 16 and each individual symptom, adjusted for age of menarche, sleep and appetite problems, physical activity levels and socioeconomic position.

**Results:**

The most prevalent symptoms were “pain” (61 %), “painful period” (10 %) and “heavy period” (33 %) at ages 16, 30 and 42, respectively. At age 16, those with severe PD were more likely to experience depression (OR: 2.92; 95% CI: 2.31, 3.70)), irritability (1.67; 1.33, 2.11), menstrual pain (1.34; 1.01, 1.80), and headaches (1.29; 1.02, 1.63). A weak association was found between severe PD at age 16 and pre-menstrual tension at age 30 (1.72; 1.01, 2.83). At age 42, those with severe PD at age 16 were more likely to experience pre-menstrual tension (1.89; 1.46, 2.44), painful periods (1.64; 1.27, 2.11), and heavy periods (1.28; 1.00, 1.62).

**Discussion:**

Menstruating females with higher levels of PD in adolescence have an increased risk of menstrual symptoms across adolescence, early and mid-adulthood. Our findings suggest the need to consider early-life psychological interventions to improve women's menstrual experiences across their reproductive years.

## Highlights

• We investigated associations between psychological distress (PD) and menstrual symptoms.
• PD at 16y was strongly associated with menstrual symptoms at ages 16, 30 and 42.
• Associations were strongest for mood-based symptoms (e.g. depression, irritability).
• Study highlights links between emotional and menstrual health over a 25-year period.
• Exploration of early-life psychological interventions for menstrual health is needed.

## Abbreviations

PD: Psychological distress
GHQ-12: 12-item General Health Questionnaire
OR: Odds ratio

## 1 Introduction

Poor menstrual experiences reoccur in traceable patterns and are one of the primary factors challenging the quality of life of girls and women every day (Bajalan et al., 2019; Guimarães and Póvoa, 2020; Ju et al., 2014). Over 14 % of girls are absent from education for one to two days each month, amounting to a staggering 140 million hours of absence annually (Pakpour et al., 2020). Not only does menstrual discomfort disrupt female students' ability to learn (Bajalan et al., 2019; Hailemeskel et al., 2016; Jeon et al., 2014), but it often continues beyond school age, going on to hinder women's performance in the workplace. As a result of their menstrual symptoms, as many as 13 % to 51 % of American women are absent from work at least once throughout their career, with 5–14 % reporting regular absences (Proctor and Farquhar, 2006; Weissman et al., 2004). Understanding the causes underlying this known, long-term negative experience and developing effective interventions to interrupt it, particularly in girls' formative years, is of key importance.

Studies have shown that psychological factors can exacerbate the discomfort caused by menstruation, influencing the way women experience and cope with their symptoms (Wang et al., 2004; Dorn et al., 2009). Psychological distress (PD) refers to non-specific symptoms of stress, anxiety and depression (Viertiö et al., 2021), and it may co-occur with physical sensations of pain, such as episodic headaches and abdominal pain (Beal et al., 2014), and simultaneously interfere with one's hormonal balance, response to stress, and pain perception (Evans et al., 2022; Hoyt and Falconi, 2015). These biological processes are intricately linked to menstrual health and may therefore worsen girls and women's experience of, and ability to cope with, the adverse side-effects of menstruation.

Existing evidence has primarily examined the impact of medically diagnosed cases of depression and anxiety on dysmenorrhea (Wang et al., 2004; Dorn et al., 2009; Rodrigues et al., 2011; Ziba, 2017; Gagua et al., 2013), defined as painful menstrual-related abdominal cramps (Bajalan et al., 2019). Whilst important, dysmenorrhea is only a subset of the diverse experiences and wide range of menstrual symptoms (up to 200 reported to date) that prevail (Campagne and Campagne, 2007). To better understand and reduce the consequences of negative menstrual experiences, it is necessary to investigate the prevalence and factors associated with a range of commonly reported menstrual symptoms, including – but not limited to – dysmenorrhea. Due to small sample sizes and a scarcity of longitudinal data, present evidence regarding the relationship between emotional and menstrual wellbeing remains inconclusive (Bajalan et al., 2019; Ju et al., 2014; Wang et al., 2004; Ziba, 2017). The majority of research is cross-sectional in nature and involves sample sizes below 500 participants (Hailemeskel et al., 2016; Dorn et al., 2009; Ziba, 2017; Gagua et al., 2013; Verma and Baniya, 2022; Pitangui et al., 2013), limiting the generalizability of findings. Without repeated prospective measurement of menstrual symptoms across the reproductive years, it is difficult to properly evaluate the complex relationship between emotional and menstrual health, especially considering the variable nature in menstrual symptoms across the reproductive life course.

As such, we aimed to examine associations between PD in adolescence (age 16) and menstrual symptoms experienced across women's life (ages 16, 30 and 42). Improving understanding of the nature and duration of this relationship can contribute to the identification of reasonable timings and targets for intervention that may promote girls and women's menstrual health. We hypothesized that girls during adolescence would be more likely to have worse affective and somatic menstrual-related symptoms both cross-sectional and into adulthood, and that associations would be stronger for mood-related symptoms.

## 2 Methods

### 2.1 Study sample

The 1970 British Cohort Study follows the lives of a large cohort, initially comprising of 17,198 individuals, born in England, Scotland or Wales between the 5th and 11th of April in 1970. To date, the cohort has been surveyed at birth and in ten subsequent waves at ages 5, 10, 16, 26, 30, 34, 38, 42, 46 and 51 (Sullivan et al., 2022). The present analysis involves data collected from self-reported questionnaires or interviews at ages 16, 30, and 42 (Sullivan et al., 2022). To be eligible for inclusion in the study sample, individuals had to have complete data on the 12-item General Health Questionnaire at age 16 and data on 1+ menstrual symptom at ages 16, 30 or 42.

### 2.2 Exposure – psychological distress (age 16 years)

The 12-item General Health Questionnaire (GHQ-12) is a validated screening tool used to measure PD (Sullivan et al., 2022). Participants were asked to report how frequently they experienced twelve unique emotional patterns on a 4-point scale (see Supplemental Table 1 for list). Negatively worded items were scored such that *“*less than usual”= 0 and “much more than usual”= 3, and reverse scoring was used for positively worded items. All items were summed to provide a total score (range: 0–36), where a higher score is indicative of increased severity in PD. The established cut-off point of 11 was used to categorize participants with low PD (<11) (Sullivan et al., 2022). Due to a high prevalence of those experiencing significant PD (≥11), a further cut-point was applied based on the median score of those experiencing PD to indicate the severity. Therefore, the three categories were: low PD (GHQ < 11), moderate PD (GHQ 11–15) and severe PD (GHQ > 15).

### 2.3 Outcome – menstrual symptoms at ages 16, 30, 42 years

**Age 16:** Participants reported whether they had started their menstrual period and if they experience unpleasant symptoms before or during their menstrual period. If the response was “yes*”* to both questions, participants were asked to specify which unpleasant symptoms they experience from the following five binary items: *pain, depression, irritability, headaches,* and *cramps*.

**Age 30:** Participants were asked “have you ever had or been told that you had a problem with your periods?*”* with dichotomous (yes/no) response options and, if so, to specify which menstrual problems from: *heavy periods, painful periods, bleeding at irregular intervals, bleeding between periods* and *pre-menstrual tension*.

**Age 42:** Participants reported whether they experienced any of the following menstrual problems in the last four years: *heavy periods*, *painful periods*, *bleeding at irregular intervals*, *bleeding between periods*, and *premenstrual tension*. All symptoms were coded as binary variables (yes/no).

### 2.4 Covariates

Covariates were measured at age 16 and chosen a priori based on associations with PD and menstrual health symptoms (Ju et al., 2014; AlQuaiz et al., 2022; Armour et al., 2019; Cholbeigi et al., 2022; De Sanctis et al., 2015; Sharma et al., 2022). *Age of menarche* and *regularity of menstrual cycle* were reported in a maternal health questionnaire where the mother of participant was asked “at what age did your teenage girl have her first menstrual period?” and “have her periods been regular in the past year*?*”. With regards to *difficulty sleeping* and *appetite problems*, the parents of participant were asked to report if their teenager struggles with each condition in two separate questions; answers were provided in a Yes/No format. To quantify the participant's *level of physical activity*, a count-based physical activity score was derived from parent-reported participation in (1) running/jogging, (2) keep fit exercises, (3) walks, (4) other forms of exercise (0–4, where 4 indicates participation in all 4 activities). Lastly, the *socioeconomic position* of the participant was categorized using the Registrar General's Social Classification of the father's occupational class: I unskilled, II partly skilled, III skilled manual, IV managerial/technical or V professional (Galobardes et al., 2007).

### 2.5 Statistical analyses

Chi-square tests and one-way ANOVAS were used to assess differences in covariates across low, moderate, and severe PD groups. Binomial logistic regression models were used to measure associations between PD at age 16 (ref: low PD) and each menstrual symptom at ages 16, 30 and 42. At age 16, outcomes included: (1) pain, (2) depression, (3) irritability, (4) headaches and (5) cramps. At ages 30 and 42, outcomes included: (1) heavy period, (2) painful period, (3) irregular bleeding, (4) bleeding between periods, and (5) pre-menstrual tension. An unadjusted and fully adjusted model was presented for each of the outcomes described above. To address missing covariate data, multiple imputation by chained equations was used to impute missing covariate data under a missing-at-random assumption. The estimates across 35 imputed datasets were combined using Rubin's rule (Rubin, 2018). The missing data ranged from 19.6 % (appetite problems) to 33.5 % (level of physical activity). Characteristics of individual missing exposure or outcome data at ages 16 were compared to the analytical sample as sensitivity analyses.

## 3 Results

### 3.1 Sample characteristics

**Fig. 1:**
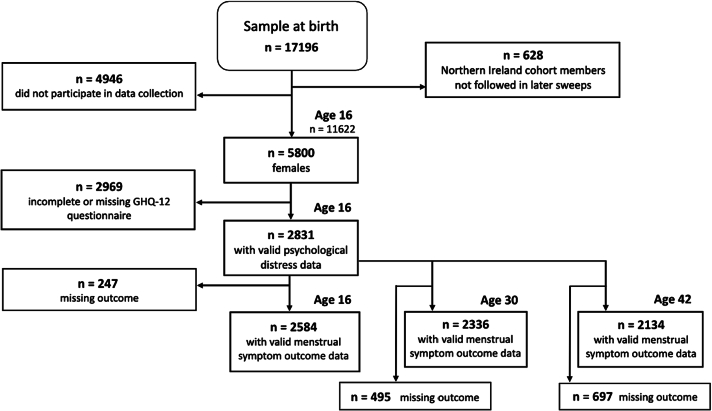
Derivation of the final analytical sample.

The analytical sample comprised 2584 participants at age 16, 2336 at age 30 and 2134 at age 42. A flowchart laying out the derivation of the included sample is presented in Fig. 1. Of 17,196 individuals included in the sample at birth, 11,622 individuals participated in a minimum of one aspect of data collection at age 16. High levels of missing GHQ-12 data were due to incomplete participation in overall data collection at age 16 due to teacher's strikes (Sullivan et al., 2022).

Table 1 describes sample characteristics across the three PD groups. Briefly, participants with severe or moderate PD were more likely to have difficulties sleeping (12.7 % vs 8.9 % vs 4.8 %; *p* < 0.001) and appetite problems (10.0 % vs 6.4 % vs 5.6 %; *p* = 0.01) compared to those with low PD. Age of menarche was slightly lower in those with severe PD (mean, SD: 12.56 ± 1.36) than those with moderate PD (12.69 ± 1.36) or low PD (12.72 ± 1.35). There were also differences within the father's occupation amongst the three PD groups (*p* = 0.03); participants with mildly or severely compromised PD were more likely to have a father in a managerial/technical or professional occupational class than those with low PD (44.7 %, 39.5 % and 35.6 %, respectively). The prevalence of experiencing irregular menstrual cycles and the level of participants' physical activeness did not differ by PD group (*p* = 0.70).

**Table 1:** Descriptive characteristics of cohort study (*n* = 2584) at age 16.

Psychological Distress (PD)				
	Low PD	Moderate PD	Severe PD	
	*n = 1700*	*n = 628*	*n = 503*	*p-value*⁎
Age of menarche (mean, SD)	12.72, 1.35	12.69, 1.36	12.56, 1.36	0.05
Irregular cycle (n(%))	255 (19.1)	113 (23.1)	82 (21.1)	0.45
Difficulty sleeping (n(%))	67 (4.8)	44 (8.9)	51 (12.7)	<0.001
Appetite problems (n(%))	77 (5.6)	32 (6.4)	40 (10.0)	0.01
Physically active (n(%))				0.70
0 (very inactive)	57 (4.9)	14 (3.5)	18 (5.5)	
1	578 (50.1)	190 (47.0)	156 (48.0)	
2	368 (31.9)	140 (34.7)	105 (32.3)	
3	119 (10.3)	48 (11.9)	33 (0.2)	
4 (very active)	31 (2.7)	12 (3.0)	13 (4.0)	
Father's occupation ((n(%))				0.03
Unskilled	31 (2.5)	11 (2.4)	13 (3.6)	
Partly skilled	121 (9.7)	35 (7.6)	26 (7.1)	
Skilled manual	485 (38.9)	147 (32.1)	126 (34.6)	
Skilled non-manual	140 (11.2)	52 (11.4)	42 (11.5)	
Managerial/technical	355 (28.5)	155 (33.8)	118 (32.4)	
Professional	88 (7.1)	50 (10.9)	26 (7.1)	

⁎ *p*-values indicate differences between groups using chi-square or one-way ANOVAs.

### 3.2 Prevalence of menstrual symptoms at age 16, 30 and 42

Table 2 provides the prevalence of symptoms at each age. At age 16, 78 % of participants reported experiencing unpleasant menstrual symptoms. Individual prevalence of symptoms ranged from 28 to 61 %, where *pain* was the most reported source of discomfort (*n* = 1579, 61 %), followed by *irritability* (*n* = 991, 38 %). At age 30, the prevalence of menstrual symptoms was substantially lower (range: 3–10 %), where *painful period* was most common (*n* = 226, 10 %). At age 42, the prevalence ranged from 10 to 33 %, where *heavy period* was the most prevalent (*n* = 707, 33 %), followed by *painful period* (*n* = 559, 26 %) and *pre-menstrual tension* (*n* = 548, 26 %).

**Table 2:** Descriptive characteristics of prevalence of menstrual symptoms at ages 16, 30 and 42.

Age of Survey	Menstrual Symptom	Prevalence n(%)
*age 16 (n* *=* *2584)*⁎	Pain	1579 (61)
	Cramps	1062 (41)
	Irritability	991 (38)
	Headaches	722 (28)
	Depression	726 (28)
*age 30 (n* *=* *2336)*	Painful Period	226 (10)
	Heavy Period	185 (8)
	Irregular Bleeding	164 (7)
	Pre-Menstrual Tension	90 (4)
	Bleeding Between Periods	70 (3)
*age 42 (n* *=* *2134)*	Painful Period	707 (33)
	Heavy Period	559 (26)
	Irregular Bleeding	548 (26)
	Pre-Menstrual Tension	285 (13)
	Bleeding Between Periods	215 (10)

⁎ 78 % of participants at age 16 reported to experience unpleasant symptoms and were thus asked to specify which of the five symptoms they experienced. The former was not explored as an outcome due to its high prevalence.

### 3.3 Associations between PD and menstrual symptoms at age 16, 30 and 42

#### 3.3.1 Menstrual symptoms at age 16

**Fig. 2:**
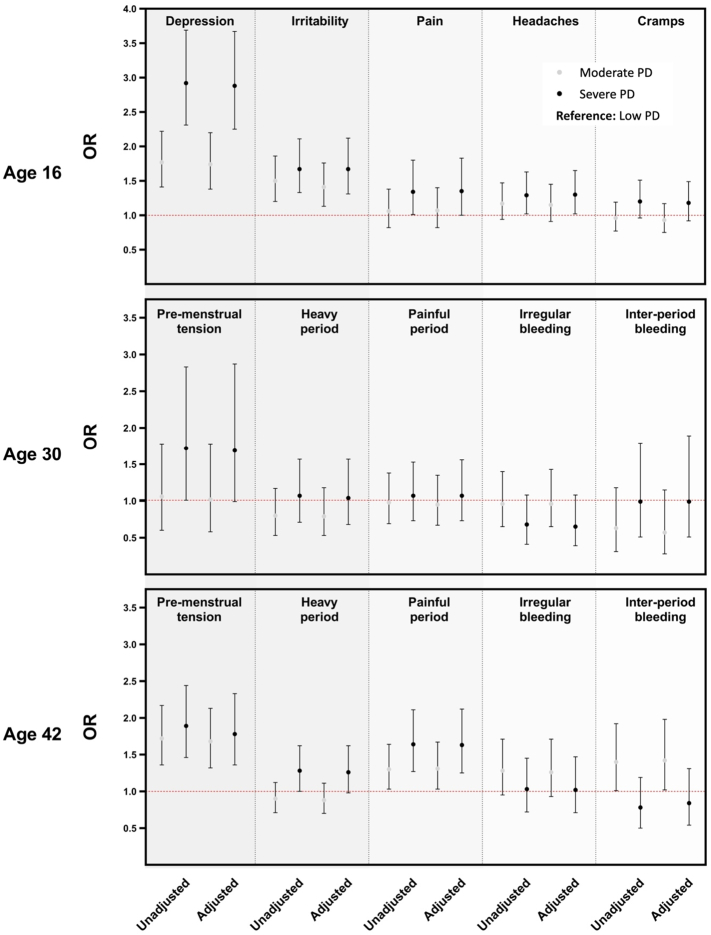
Odds of experiencing menstrual symptoms at ages 16, 30 and 42 depending on PD status at age 16 (reference group: low PD at age 16). Adjusted models include age of menarche, sleep and appetite problems, physical activity levels and socioeconomic status [OR: Odds ratio]).

PD at age 16 was associated with greater odds of reporting unpleasant menstrual symptoms (see Fig. 2), with strongest associations for *depression* and *irritability*. For example, in unadjusted models, those with moderate and severe PD had 1.77 (95 % CI: 1.41, 2.22) and 2.92 (2.31, 3.70) times higher odds of *depression* during menstruation than those with low PD. Similarly, those with moderate and severe PD had higher odds of *irritability* than those with low PD (1.50 (1.20, 1.86); 1.67 (1.33, 2.11), respectively). Those with severe PD had 1.29 (1.02, 1.63) greater odds of having *period-related headaches*, although there was no increased risk in those with moderate PD. All associations persisted after adjustment for relevant covariates. Finally, there was no evidence of an association between PD and *cramps*, with some evidence to suggest that those with severe PD were more likely to have *period-related pain* (adjusted: 1.35 (1.00,1.83)).

#### 3.3.2 Menstrual symptoms at age 30

No associations were observed between PD at age 16 and *heavy period*, *painful period*, *irregular bleeding*, and *bleeding between periods* at age 30. In unadjusted models, those with severe PD at age 16 had 1.72 (1.01, 2.83) higher odds of experiencing *pre-menstrual tension* than those with low PD, with very little change in adjusted models (1.69 (0.99, 2.87)).

#### 3.3.3 Menstrual symptoms at age 42

PD at age 16 was associated with greater risk of having unpleasant menstrual symptoms at age 42, with strongest associations for *pre-menstrual tension* and *painful period*. In unadjusted models, those with moderate and severe PD had 1.72 (1.36, 2.17) and 1.89 (1.46, 2.44) higher odds of having *pre-menstrual tension* and 1.30 (1.03, 1.64) and 1.64 (1.27, 2.11) greater odds of having *painful periods*, respectively. Associations persisted after adjustment for covariates. Those with moderate PD had 1.40 (1.01, 1.92) higher odds of *bleeding between periods*, in unadjusted models, but no association was observed in those with severe PD (0.78 (0.50, 1.19)). Those with severe PD in adolescence were more likely to experience *heavy periods* (adjusted model: 1.26 (0.98, 1.62). Finally, there was no evidence of an association between PD and *irregular bleeding*.

#### 3.3.4 Comparison of characteristics of analytical sample and those missing GHQ-12 or menstrual data at age 16

Supplementary Table 3 outlines characteristics of those with missing data at age 16 compared to the analytical sample. Briefly, those missing data due to incomplete GHQ-12 or menstrual cycle data at age 16 had an older mean age at menarche, were slightly less active and were more likely to have a father from an unskilled/partly skilled occupation. There were no differences in the prevalence of irregular cycles, difficulty sleeping or appetite problems. Additionally, there were minimal differences in menstrual cycle symptoms at age 16 between the analytical sample and those with missing GHQ-12 data only; with slightly lower prevalence of cramps in those with missing data (46 % vs 53 %; *p* < 0.001).

## 4 Discussion

### 4.1 Key findings and comparison to literature

In a large birth cohort study of individuals followed across the life course, we found that higher PD at age 16 was associated with a higher risk of reporting adverse menstrual symptoms in adolescence and during early and mid-adulthood. Associations were strongest for mood-related symptoms, including *depression* and *irritability* at age 16 and *pre-menstrual tension* at age 30 and 42. These findings highlight the need to consider mental health as both an acute and long-term indicator, contributor, and potential determinant of women's menstrual experiences and overall health and wellbeing.

The positive association between increased PD and higher odds of reporting mood-related symptoms is consistent with previous cross-sectional research. Past studies have found that psychological disorders can exacerbate the number and severity of menstrual symptoms girls and women experience (Pakpour et al., 2020; Dorn et al., 2009; Beal et al., 2014; AlQuaiz et al., 2022). In a cross-sectional study of 154 girls (mean age: 15.4 years), subjects with more symptoms of depression and anxiety experienced a greater number of unspecified menstrual symptoms (Dorn et al., 2009). A similar study that integrated a short follow-up component of three annual surveying visits found comparable results (Beal et al., 2014). Interview-based evidence from a larger sample (*n* = 1831 women, age range: 18–50 years) highlight an association between increased severity of psychological distress and six domains directly related to pre-menstrual syndrome. This association was strongest with anxiety/mood menstrual-related symptoms (odds ratio: 2.75(1.92,3.94)) (AlQuaiz et al., 2022). Younger women - those aged 18–30 years - reported anxiety/mood menstrual-related symptoms more frequently than women aged 30–50 years (AlQuaiz et al., 2022). This is consistent with the results found in the present study where *depression* during menstruation was the most prevalent symptom at age 16, and associations were strongest between PD and the depression symptom. Distinctive to these studies, our findings provide novel insights into the potential life-course consequences of severe levels of PD, by demonstrating sustained associations into midlife.

### 4.2 Hypothesized pathways of association

It remains unclear whether the association between PD and menstrual health is purely correlational or if there is an element of causality involved. Physiological evidence in the literature supports the plausibility of the latter. Prostaglandins are important compounds with hormone-like effects that help regulate pain perception and bodily inflammation (Smith, 2018). Release and circulation of prostaglandins must be maintained in equilibrium to ensure that physiological processes such as uterine contractions during menstruation are carried out effectively. Experience of intense emotions can lead to the release of stress hormones (i.e., cortisol and adrenaline), which stimulate the production of prostaglandins (García-Bueno et al., 2008). Reoccurring incidences of psychological distress can lead to destabilizing levels of prostaglandins circulating the body, which could interfere with the body's mediation of abdominal or uterine inflammation and sensitivity to pain (Pakpour et al., 2020; Smith, 2018). Consequently, those experiencing significant PD may therefore explore a greater intensity of unpleasant menstrual symptoms.

Psychological distress may also predispose individuals to engage in coping mechanisms such as smoking, poor eating habits, and sedentary outlets (Leary et al., 2022). These behaviors contribute to poor health lifestyles that, if sustained, could also exacerbate menstrual symptoms (Hightower, 1997). Further research is required to formally test if prostaglandins, stress hormones and compensatory behavior change mediate this association.

Severe PD was more strongly associated with mood-related symptoms (i.e., depression, irritability, and pre-menstrual tension) when compared to somatic menstrual outcomes (i.e., heavy period and irregular bleeding). This was observed at all ages, although it was particularly evident at 16 and 42. Greater PD may increase one's sensitivity to discomfort such that one is more likely to recognize and report unpleasant menstrual symptoms (Evans et al., 2022). Since the experience of mood-related symptoms can be argued to be of more subjective nature than their somatic counterparts (Pakpour et al., 2020), it is plausible that negative menstrual experiences are heightened for the former. It was notable that strong associations persisted at age 42. Poor emotional wellbeing at adolescence could be sustained into midlife or could predispose the participants to greater emotional sensitivity, such that emotional imbalances are more easily triggered during sensitive time points. As women begin to approach perimenopause and experience hormonal fluctuations – particularly in the production of luteinizing hormone and follicle stimulating hormone –, this can often result in increased irregularity of menstrual cycles and variability in symptoms (Hoyt and Falconi, 2015). This transition brings uncertainty which can intensify negative emotional responses and shift one's perception and sensitivity in the reporting of unpleasant menstrual symptoms (Woods et al., 2023).

At age 30, severe PD was weakly associated with *pre-menstrual tension* only. Around this age, women are typically in their childbearing years and may therefore experience disrupted menstrual cycles, due to pregnancy or hormonal contraceptives, and therefore have a poorer recollection of menstrual symptoms. This is consistent with the decrease in the prevalence of unpleasant menstrual symptoms reported (see Table 1**)**. Furthermore, unpleasant symptoms could temporarily cease due to the reasons noted above, to stabilization of menstrual experiences, or due to being unnoticed and thus under-reported.

Considering these insights and plausible pathways, it is important to acknowledge that PD may serve as an indicator of underlying depressive conditions, potentially overlapping with menstrual-related depressive symptoms. This overlap could exacerbate the sensitivity of the association between psychological distress and menstrual symptoms. Hence, further research is needed to disentangle the complex associations between clinical depression and exacerbated menstrual-related depressive symptoms.

### 4.3 Strengths and limitations

Key strengths of this study include the large age-homogenous sample size, ascertainment of multiple menstrual symptoms and the longitudinal study design. The majority of previous studies are limited to samples below 500 participants and informed by a single time point, which makes it difficult to contextualize their temporal relevance throughout the reproductive period. The present findings help to fill this gap as they are informed by a cohort of over 2000 individuals with menstrual symptom data from over a 25-year period (i.e., from age 16 to 42).

Study limitations include the binary menstrual health questions that were unable to capture the severity of symptoms nor the extent to which symptoms affect the participant's ability to carry out daily life activities. This information would be of value since it could help to understand which symptoms require most attention to improve women's menstrual experiences and their quality of life. Ascertainment of menstrual symptoms was not consistent across the three time points. Although reflective of secular understandings of menstrual symptoms at data collection timepoints, these differences limit the ability to infer age-dependent patterns about specific symptoms. Finally, the sample size was reduced due to a teacher strike at age 16 which impacted overall participation rates, individual-level missing or incomplete GHQ and menstrual health data across the three ages as well as overall attrition in the study (see Fig. 1). However, compared to low sample sizes of previous research, the relatively large sample is still viewed as a major strength of the data and there were minimal differences between the analytical sample and those excluded due to missing data.

### 4.4 Implications and future research

This paper provides novel insights into the acute and long-term relationship between mental health and unfavorable menstrual experiences, that can guide future research and ultimately support the improvement of menstrual health experiences for girls and women worldwide. To our knowledge, this is the first study to examine long-term associations between mental and menstrual health over a 25-year period. Additionally, the age-homogeneity of the sample removes any age-related differences that are typically observed in menstrual symptom prevalence. Results from this study underscore the need for further research to unravel the intricate interplay between women's reproductive stage and the emotional factors affecting women's health and strengthen support for the investment in adolescent menstrual health interventions. Targeted research and evidence-based interventions, particularly in girls' formative years, offer the potential to alleviate the burden of unpleasant menstrual symptoms and to mitigate possible long-term consequences of severe psychological distress on women's reproductive health.

## Sources of funding

JMB is supported through a 10.13039/501100000274British Heart Foundation grant (SP/F/20/150002). JJM is supported through a 10.13039/501100000265Medical Research Council grant (MR/N013867/1). CM is supported through the UCL Laidlaw Research and Leadership Programme.

## Ethics approval and consent to participate

Informed consent was obtained from all participants. Participants were sent written materials which provided a full explanation of what taking part in the research would involve. At the beginning of the visit, interviewers/nurses checked that participants had read and understood the materials and were willing to proceed. Ethics were approved internally at age 16 (1986), by the London Multicentre Research Ethics Committee at age 30 (2000, 98/2/120) and by the London-Central Ethics Committee at age 42 (2012, 11/LO/1560).

## Implications and contribution statement

The study provides novel insights into long-term associations between emotional and menstrual health and highlight the potential need for early-life psychological interventions to reduce adverse menstrual outcomes across all reproductive years.

## CRediT authorship contribution statement

**C. Martins:** Writing – review & editing, Writing – original draft, Visualization, Methodology, Investigation, Funding acquisition, Formal analysis, Conceptualization. **J.J. Mitchell:** Writing – review & editing, Visualization, Supervision, Formal analysis. **M. Hamer:** Writing – review & editing, Resources, Funding acquisition, Conceptualization. **J.M. Blodgett:** Writing – review & editing, Supervision, Project administration, Methodology, Funding acquisition, Conceptualization.

## Declaration of competing interest

The authors declare that the research was conducted in the absence of any competing or conflicts of interest.

## Data Availability

All datasets used to carry out the study are available at UK Data Service to boo field researchers. Age 16 doi: https://doi.org/10.5255/UKDA-SN-3535-6; Age 30 doi: https://doi.org/10.5255/UKDA-SN-5558-3; Age 42 doi: https://doi.org/10.5255/UKDA-SN-7473-3.

## Appendix A Supplementary data

Supplementary tables Image 1
