# Supplementary material for: Associations between psychological distress in adolescence and menstrual symptoms across life: Longitudinal evidence from the 1970 British Cohort Study
Source: J Affect Disord. 2024 Jun 1;354:712–8. doi: 10.1016/j.jad.2024.03.069 (PMC11752839; doi:10.1016/j.jad.2024.03.069)
Supplement: Supplementary file 1 — Supplementary tables [file mmc1.docx]

**Supplemental Table 1** – GHQ-12 items used to measure the PD of participants.

| **Item Number** | **Statement** |
| --- | --- |
|  |  |
| *1* | Able to concentrate on what I am doing |
| *2* | Capable of making decisions about things |
| *3* | Able to face up to my problems |
| *4* | Reasonably happy all things considered |
| *5* | Able to enjoy normal daily activities |
| *6* | Felt I am playing a useful part in things |
| *7* | Lost much sleep over worry |
| *8* | Felt constantly under strain |
| *9* | Felt I couldn't overcome my difficulties |
| *10* | Been feeling unhappy and depressed |
| *11* | Been losing confidence in myself |
| *12* | Thinking of myself as a worthless person |
|  |  |

**Supplemental Table 2** - Menstrual symptoms present at ages 16, 30 and 42 depending on PD status at age 16 (reference group: low PD). Adjusted models include age of menarche, sleep and appetite problems, physical activity levels and socioeconomic status [OR: odds ratio].

| **--- Age 16 ---** | | **Depression** (n=2018) | | | | **Irritability** (n=2018) | | | | **Pain** (n=2018) | | | | **Headaches** (n=2018) | | | | **Cramps** (n=2018) | | | |  |
| --- | --- | --- | --- | --- | --- | --- | --- | --- | --- | --- | --- | --- | --- | --- | --- | --- | --- | --- | --- | --- | --- | --- |
|  |  | *OR* | *95% CI* | | *p-value* | *OR* | *95% CI* | | *p-value* | *OR* | *95% CI* | | *p-value* | *OR* | *95% CI* | | *p-value* | *OR* | *95% CI* | | *p-value* |  |
|  |  |  |  |  |  |  |  |  |  |  |  |  |  |  |  |  |  |  |  |  |  |  |
| **Model 1** (unadjusted) | Low PD |  |  |  |  |  |  |  |  |  |  |  |  |  |  |  |  |  |  |  |  |  |
|  | Moderate PD | 1.77 | 1.41, 2.22 | | 0.00 | 1.50 | 1.20, 1.86 | | 0.00 | 1.06 | 0.82, 1.38 | | 0.64 | 1.17 | 0.94, 1.47 | | 0.16 | 0.96 | 0.77, 1.19 | | 0.68 |  |
|  | Severe PD | 2.92 | 2.31, 3.69 | | 0.00 | 1.67 | 1.33, 2.11 | | 0.00 | 1.34 | 1.01, 1.80 | | 0.05 | 1.29 | 1.02, 1.63 | | 0.04 | 1.20 | 0.96, 1.51 | | 0.11 |  |
| **Model 2** (adjusted) | Low PD |  |  |  |  |  |  |  |  |  |  |  |  |  |  |  |  |  |  |  |  |  |
|  | Moderate PD | 1.74 | 1.38, 2.20 | | 0.00 | 1.41 | 1.13, 1.76 | | 0.00 | 1.07 | 0.82, 1.40 | | 0.61 | 1.15 | 0.91, 1.45 | | 0.23 | 0.93 | 0.75, 1.17 | | 0.54 |  |
|  | Severe PD | 2.88 | 2.25, 3.67 | | 0.00 | 1.67 | 1.31, 2.12 | | 0.00 | 1.35 | 1.00, 1.83 | | 0.05 | 1.30 | 1.02, 1.65 | | 0.04 | 1.18 | 0.92, 1.49 | | 0.19 |  |
| **--- Age 30 ---** | | **Pre-Menstrual Tension** (n=2336) | | | | **Heavy Period** (n=2336) | | | | **Painful Period** (n=2336) | | | | **Irregular Bleeding** (n=2336) | | | | **Bleeding Between Periods** (n=2336) | | | |  |
|  |  | *OR* | *95% CI* | | *p-value* | *OR* | *95% CI* | | *p-value* | *OR* | *95% CI* | | *p-value* | *OR* | *95% CI* | | *p-value* | *OR* | *95% CI* | | *p-value* |  |
| **Model 1** (unadjusted) | Low PD |  |  |  |  |  |  |  |  |  |  |  |  |  |  |  |  |  |  |  |  |  |
|  | Moderate PD | 1.06 | 0.60, 1.78 | | 0.84 | 0.80 | 0.53, 1.17 | | 0.26 | 0.98 | 0.69, 1.38 | | 0.93 | 0.96 | 0.65, 1.40 | | 0.84 | 0.63 | 0.31, 1.18 | | 0.17 |  |
|  | Severe PD | 1.72 | 1.01, 2.83 | | 0.04 | 1.07 | 0.71, 1.57 | | 0.74 | 1.07 | 0.73, 1.53 | | 0.72 | 0.68 | 0.41, 1.08 | | 0.12 | 0.99 | 0.51, 1.79 | | 0.97 |  |
| **Model 2** (adjusted) | Low PD |  |  |  |  |  |  |  |  |  |  |  |  |  |  |  |  |  |  |  |  |  |
|  | Moderate PD | 1.02 | 0.58, 1.78 | | 0.94 | 0.79 | 0.53, 1.18 | | 0.26 | 0.95 | 0.67, 1.35 | | 0.79 | 0.96 | 0.65, 1.43 | | 0.84 | 0.57 | 0.28, 1,15 | | 0.12 |  |
|  | Severe PD | 1.69 | 0.99, 2.87 | | 0.05 | 1.04 | 0.68, 1.57 | | 0.86 | 1.07 | 0.73, 1.56 | | 0.73 | 0.65 | 0.39, 1.08 | | 0.09 | 0.99 | 0.51, 1.89 | | 0.97 |  |
| **--- Age 42 ---** | | **Pre-Menstrual Tension** (n=2134) | | | | **Heavy Period** (n=2134) | | | | **Painful Period** (n=2134) | | | | **Irregular Bleeding** (n=2134) | | | | **Bleeding Between Periods** (n=2134) | | | |  |
|  |  | *OR* | *95% CI* | | *p-value* | *OR* | *95% CI* | | *p-value* | *OR* | *95% CI* | | *p-value* | *OR* | *95% CI* | | *p-value* | *OR* | *95% CI* | | *p-value* |  |
| **Model 1** (unadjusted) | Low PD |  |  |  |  |  |  |  |  |  |  |  |  |  |  |  |  |  |  |  |  |  |
|  | Moderate PD | 1.72 | 1.36, 2.17 | | 0.00 | 0.90 | 0.71, 1.12 | | 0.34 | 1.30 | 1.03, 1.64 | | 0.03 | 1.28 | 0.95, 1.71 | | 0.10 | 1.40 | 1.01, 1.92 | | 0.04 |  |
|  | Severe PD | 1.89 | 1.46, 2.44 | | 0.00 | 1.28 | 1.00, 1.62 | | 0.05 | 1.64 | 1.27, 2.11 | | 0.00 | 1.03 | 0.72, 1.45 | | 0.86 | 0.78 | 0.50, 1.19 | | 0.27 |  |
| **Model 2** (adjusted) | Low PD |  |  |  |  |  |  |  |  |  |  |  |  |  |  |  |  |  |  |  |  |  |
|  | Moderate PD | 1.68 | 1.32, 2.13 | | 0.00 | 0.88 | 0.70, 1.11 | | 0.28 | 1.31 | 1.03, 1.67 | | 0.03 | 1.26 | 0.93, 1.71 | | 0.13 | 1.42 | 1.02, 1.98 | | 0.04 |  |
|  | Severe PD | 1.78 | 1.36, 2.33 | | 0.00 | 1.26 | 0.98, 1.62 | | 0.07 | 1.63 | 1.25, 2.12 | | 0.00 | 1.02 | 0.71, 1.47 | | 0.89 | 0.84 | 0.54, 1.31 | | 0.44 |  |

**Supplemental Table 3 –** Characteristics of analytical sample at age 16 compared to those excluded due to missing GHQ or menstrual data

|  | Analytical sample (n=2584) | Age 16 participants lost due to incomplete GHQ-12 data (n=2969) | Age 16 participants lost due to incomplete  menstrual data (n=247) | p-value |
| --- | --- | --- | --- | --- |
|  |  |  |  |  |
| BASIC CHARACTERISTICS |  |  |  |  |
| Age of menarche (mean, SD) | 12.68, 1.34 | 12.80, 1.38 | 12.78, 1.49 | 0.01 |
| Irregular cycle (Y, %, (n)) | 21% (421) | 19% (399) | 18% (29) | 0.29 |
| Difficulty sleeping (Y, %, (n)) | 7% (155) | 6% (139) | 4% (7) | 0.12 |
| Appetite problems (Y, %, (n)) | 6% (136) | 8% (186) | 7% (13) | 0.07 |
| Physically active (0-4, %, (n)) |  |  |  | 0.02 |
| *0 (very inactive)* | 5% (81) | 7% (117) | 6% (8) |  |
| *1* | 49% (851) | 51% (844) | 51% (73) |  |
| *2* | 33% (565) | 28% (466) | 33% (48) |  |
| *3* | 11% (187) | 11% (174) | 9% (13) |  |
| *4 (very active)* | 3% (54) | 2% (39) | 1% (2) |  |
| Father’s occupation (type, %, (n)) |  |  |  | <0.001 |
| *Unskilled or Partly skilled* | 11% (218) | 13% (192) | 14% (19) |  |
| *Skilled manual or Skilled non-manual* | 50% (930) | 54% (772) | 44% (62) |  |
| *Managerial/technical or Professional* | 39% (722) | 33% (463) | 42% (59) |  |
|  |  |  |  |  |
| MENSTRUAL OUTCOMES (%, (n)) |  |  |  |  |
| Pain | 78% (1579) | 79% (512) |  | 0.58 |
| Cramps | 53% (1062) | 46% (295) |  | <0.01 |
| Irritability | 49% (991) | 47% (306) |  | 0.49 |
| Headaches | 36% (722) | 37% (241) |  | 0.49 |
| Depression | 36% (726) | 38% (243) |  | 0.46 |
